# Discovery of two new *Cortinarius* species in Southern China

**DOI:** 10.3389/fmicb.2025.1558935

**Published:** 2025-05-09

**Authors:** Yuxiao Dang, Mengjia Zhu, Nemat O. Keyhani, Ziyi Wu, Chenghao Qiu, Jinming Xiong, Huili Pu, Longbing Lin, Chengjie Xiong, Zhiying Zhao, Minghai Zheng, Taichang Mu, Yongsheng Lin, Yanbin Huang, Xin Yuan, Chunjin Qiu, Xiaohong Ji, Junzhi Qiu, Yuxi Chen

**Affiliations:** ^1^State Key Laboratory of Agricultural and Forestry Biosecurity, College of Life Sciences, Fujian Agriculture and Forestry University, Fuzhou, China; ^2^Department of Biological Sciences, University of Illinois, Chicago, IL, United States; ^3^XiangYa School of Public Health, Central South University, Changsha, China; ^4^Institute of Microbiology, Chinese Academy of Sciences, Beijing, China; ^5^Bureau of Fujian Junzifeng National Nature Reserve, Sanming, China; ^6^Bureau of Fujian Longqishan National Nature Reserve, Sanming, China; ^7^Putian Institute of Agricultural Sciences, Putian, China; ^8^College of Pharmacy and Life Sciences, Jiujiang University, Jiujiang, China; ^9^College of Bee Science and Biomedicine, Fujian Agriculture and Forestry University, Fuzhou, China

**Keywords:** Agaricales, morphological, new species, phylogenetic analyses, taxonomy

## Abstract

**Introduction:**

*Cortinarius* are globally distributed mushrooms, all of whom form mycorrhizae, and are characterized by rust-brown to brownish-red spores. The high species richness of the genus results in significant morphological diversity overall, alongside high similarity among closely related species, leading many taxonomists to concentrate their studies on specific infrageneric groups.

**Methods:**

Specimens were noted and collected from Fujian Province, China. Genomic DNA was extracted from specimens and the nucleotide sequences of two loci (ITS AND nr LSU) were determined and used to construct phylogenetic trees. Microscopic features were observed using an optical microscope. Fluorescence reactions were examined using a fluorescence microscope.

**Results:**

We describe two new species of *Cortinarius*, *C. griseoaurantinus* (subgenus *Leprocybe*) and *C. yonganensis* (subgenus *Dermocybe*), from Fujian Province, Southern China. Comprehensive morphological descriptions, color photographs of fresh basidiomata, photographs of microscopic characters, and phylogenetic trees are provided.

**Discussion:**

These data identify new species of *Cortinarius*, enhancing the understanding of the genus and the ecological relationships. The developed keys provide a reference for the further analysis of the evolution and geographic distribution of these fungi.

## 1 Introduction

The genus *Cortinarius* (Pers.) Gray (commonly known as cortinar or webcap) belongs to the family Cortinariaceae and the order Agaricales, within Basidiomycota. It is one of the most diverse genera of mushroom-forming fungi, comprising thousands of species found worldwide (Moser et al., 1975; Garnica et al., 2016; Liimatainen et al., 2017; Varga et al., 2019; Liimatainen et al., 2020; Dima et al., 2021). According to previous reports, extracts from certain *Cortinarius* species are harmful to human health. For example, a type of sterol from *Cortinarius xiphidipu*s has shown cytotoxic activity (Torres et al., 2017); orellanine from *Cortinarius orellanus* can cause acute nephritis (Nusair et al., 2023); emodin from *Cortinarius sanguineus* has shown cytotoxic and skin-sensitizing effects (Yli-Öyrä et al., 2024); *Cortinarius* species have also been reported to cause myocarditis (Balice et al., 2024). Nevertheless, some *Cortinarius* species are edible, such as *Cortinarius purpurascens* (Bai et al., 2013) and *Cortinarius caerulescens* (Song et al., 2022). However, the high morphological similarity between certain species within the genus makes it challenging to distinguish them based solely on appearance. Moreover, the edibility status of the majority of species remains largely undocumented and requires further investigation. Therefore, currently, there is no reliable method to determine the toxicity of *Cortinarius* species based solely on its morphological characteristics. All members of the genus are ectomycorrhizal fungi, forming symbiotic associations with a wide variety of vascular plants across the majority of ecosystems, from the tropics to the Arctic (Moser et al., 1975). A defining characteristic of *Cortinarius* species is their rusty-brown spore print, which is a key diagnostic feature for identification. Some species (e.g., *C. sanguineus*) are notably colorful and have been used as natural sources of pigment dyes (Frøslev et al., 2007; Niskanen et al., 2009; Liimatainen et al., 2014a, b; Zhang et al., 2023; Long et al., 2024). Due to the genus’s extensive species richness, there is considerable morphological diversity overall, along with high similarity between closely related species. Consequently, many taxonomists have focused on specific infrageneric groups in their studies (Brandrud, 1998; Niskanen et al., 2016; Pastor et al., 2019; Soop et al., 2019; Liimatainen et al., 2022; Zhang et al., 2023; Fan et al., 2024; Long et al., 2024).

Historically, *Cortinarius* was the only genus recognized in the family Cortinariaceae. However, recent phylogenomic studies have divided it into 10 separate genera: *Aureonarius* Niskanen & Liimat., *Austrocortinarius* Niskanen & Liimat., *Calonarius* Niskanen & Liimat., *Cortinarius* (Pers.) Gray., *Cystinarius* Niskanen & Liimat., *Hygronarius* Niskanen & Liimat., *Mystinarius* Niskanen & Liimat., *Phlegmacium* Niskanen & Liimat., *Thaxterogaster* Singer., and *Volvanarius* Niskanen & Liimat., seven of which—*Aureonarius*, *Austrocortinarius*, *Calonarius*, *Cystinarius*, *Hygronarius*, *Mystinarius*, *and Volvanarius—*were newly described (Liimatainen et al., 2022). This division has been contested by Gallone et al. (2024), who reanalyzed the phylogenomic dataset and identified substantial conflicts between gene trees and species trees. This latter work showed that the phylogenetic relationships within the genus *Cortinarius* remain unresolved, with phylogenomic hypotheses displaying short and weakly supported backbone branches. Of the 10 proposed genera, only four—*Cortinarius*, *Phlegmacium*, *Aureonarius*, *and Thaxterogaster—*were confirmed to be monophyletic, leaving the overall relationships and branching order among groups uncertain (Gallone et al., 2024). To further complicate the issue, within *Cortinarius*, proposed subgenera include *Cortinarius*, *Camphorati*, *Dermocybe*, *Illumini*, *Infracti*, *Iodolentes*, *Leprocybe*, *Myxacium*, *Orellani*, *Paramyxacium*, and *Telamonia* (Liimatainen et al., 2022; Fan et al., 2024).

The *Cortinarius* subgenus *Leprocybe* was first described by Moser (1969) and was further revised by Niskanen et al. (2009) and Liimatainen et al. (2022). According to the latter, species of the *Cortinarius* subgenus *Leprocybe* share a common ancestor; however, some reported nodal support values are low (below 70%). This may be due to different phylogenetic analysis methods (e.g., concatenated analyses and multispecies coalescent model analyses) yielding varying conclusions, which increases uncertainty in classification. Currently, it is not possible to determine the monophyly of the *Cortinarius* subgenus *Leprocybe*. Morphologically, members of the *Leprocybe* subgenus include species with small to medium-sized (occasionally large) basidiocarps, characterized by a typical leprocyboid/dermocyboid or sequestrate appearance, with a dry pileus and stipe, and displaying a yellow, red, or greenish-olive hue. Certain parts of the basidiocarps also possess fluorescent properties (Moser et al., 1975; Liimatainen et al., 2014b; Vellinga et al., 2015; Hong et al., 2024).

The subgenus *Dermocybe* (common name: skin-heads) within *Cortinarius* is globally distributed and includes members known for their vibrant and diverse colors, such as the vivid green of *C. austrovenetus* and the blood-red sheen of the hymenophore of *C. semisanguineus*. These fungi are also characterized by their non-hygrophanous, dry caps, dry cylindrical non-bulbous stems, and ecological adaptability. The lethal web caps, regarded as some of the world’s most poisonous mushrooms, belong to this group. Members of *Dermocybe* can be distinguished from other subgenera by their small to medium-sized basidiomata, bright colors, and typically slender stipes (Matheny et al., 2014; Liimatainen et al., 2022). Many species within this group contain unique pigments that have garnered significant attention in ecological studies and biochemical analyses (Matheny et al., 2014; Liimatainen et al., 2022). Anthraquinone is present in the basidiomata of the subgenus *Dermocybe* and exhibits diverse biological activities. These activities are utilized in crop protection and serve as sustainable, bio-based colorants for textiles, paints, electronics, and cosmetics industries (Fiala et al., 2025; Jetha et al., 2025).

During fieldwork in Fujian Province, China, two potentially new species of *Cortinarius* were discovered. A detailed morphological examination, along with molecular sequencing and phylogenetic analyses of the nuclear ribosomal internal transcribed spacer (ITS) and nuclear ribosomal large subunit (nrLSU) sequences, confirmed that the specimens represent two new species. They were named *Cortinarius griseoaurantinus* (subgenus *Leprocybe*) and *Cortinarius yonganensis* (subgenus *Dermocybe*). The macro- and micro-morphological features of the two new species, accompanied by photographs, are provided in this study. These findings enhance our understanding of the diversity within the *Cortinarius* genus.

## 2 Materials and methods

### 2.1 Collection and morphological analyses

Specimens of *Cortinarius griseoaurantinus* were collected from Mingxi and Mount Wuyi, Fujian Province, in 2023. Specimens of *Cortinarius yonganensis* were collected from Yong’an City, Fujian Province, in 2023. Images of fresh fruiting bodies were captured using a Canon EOS 6D Mark II camera. The samples were dried at the Fujian Agriculture and Forestry University Fungal Herbarium (HMFAFU) and then deposited in the Fungarium (HMAS) of the Institute of Microbiology, Chinese Academy of Sciences (CAS). Macromorphological descriptions were based on field notes and color photos of basidiocarps. Descriptions of the relevant color codes followed (Kornerup and Wanscher, 1981).

After rehydrating dried tissues in 5% KOH and staining them with a 1% Congo red solution, microscopic features such as basidia and spores were observed using an optical microscope (Leica DM2500). Following the rehydration of dried tissues in 5% KOH, fluorescence reactions were examined using a fluorescence microscope (Carl Zeiss Axio Imager A2). At least 40 basidiospores were analyzed. The dimensions of basidiospores are presented in the notation (a) b-c-d (e). The range b-d includes a minimum of 90% of the measured values. Mean value, i.e., c is provided. Extreme values, i.e., a or e, are provided in parentheses. Q denotes the “length/width ratio” of a basidiospore in a side view; Q indicates the average Q of all basidiospores ± the sample standard deviation.

### 2.2 DNA extraction, PCR amplification, and sequencing

Genomic DNA was extracted from dry specimens (10–50 mg) using the D3390 Fungal DNA Mini KIT (OMEGA, United States), following the manufacturer’s instructions. To amplify specific genetic regions, the primer pair ITS5/ITS4 (White et al., 1990) was utilized for the internal transcribed spacer (ITS) region of the nuclear ribosomal repeat unit, and the primer pair LROR/LR5 was used for the nuclear ribosomal large subunit (nrLSU) region of the nuclear ribosomal repeat unit. PCR reactions (25 μL total volume) were prepared with 1 μL of DNA solution, 12.5 μL of 2 × PCR mix enzyme, 9.5 μL of sterile deionized water, and 1 μL of each primer.

PCR amplifications were conducted as described. The conditions were set as follows: for ITS amplification, an initial denaturation at 95°C for 3 min, followed by 30 cycles at 95°C for 30 s, 55°C for 45 s, and 72°C for 1 min, and a final extension at 72°C for 10 min (Hong et al., 2024); for LSU amplification, an initial denaturation at 94°C for 1 min, followed by 35 cycles at 94°C for 30 s, 55°C for 1 min, and 72°C for 1.5 min, and a final extension at 72°C for 10 min (Fan et al., 2024). PCR products were subjected to electrophoresis on 1% agarose gel stained with a nucleic acid dye. Subsequently, the samples were sent for sequencing at Fuzhou Biosune Company using the same primers. All newly generated sequences for ITS and nrLSU in this study have been submitted to GenBank.

### 2.3 Alignment and phylogenetic analyses

Initial BLAST searches^1^ using the derived ITS and nrLSU sequences confirmed that the isolate is grouped within *Cortinarius*. Sequences for the final dataset were selected from previously published literature (Fan et al., 2024; Hong et al., 2024) and GenBank (Table 1). The quality of the sequences was considered during the selection process for phylogenetic analyses. *Hebeloma cylindrosporum* was selected as the outgroup. Sequence alignment and adjustment of ITS and nrLSU sequences were performed separately using MEGA7 software (Kumar et al., 2016). The PhyloSuite software (Zhang et al., 2020) was then employed to concatenate the sequences and conduct Maximum Likelihood (ML) and Bayesian Inference (BI) analyses. Detailed specimen information and GenBank accession numbers used in this study are provided in Table 1. Sequence selection was based on previously described criteria (Fan et al., 2024; Hong et al., 2024).

**Table 1:** Species and specimens of *Cortinarius* and *Hebeloma* used for the molecular phylogenetic analyses.

Species	Sample no.	Country	GenBank	
			ITS	nrLSU
*Cortinarius anomalus*	CA3	Norway	KC842425	KC842495
*C. anomalus*	CFP1154	Sweden	KX302224	-
*C. anomalus*	TUB011883	Germany	AY669645	AY669645
*C. barlowensis*	JFA13140	North America	FJ717554	FJ717554
*C. bolaris*	3,861	Canada	KJ705110	-
*C. bolaris*	CFP1008	Sweden	KX302233	-
*C. bolaris*	T40	Norway	KC842426	KC842496
*C. bolaris*	TUB0118524	Germany	AY669596	AY669596
*C. calaisopus*	PDD103678	New Zealand	KF727395	-
*C. calaisopus*	PDD94050	Dunedin	NR157880	NG068868
*C. camphoratus*	SMI193	Canada	FJ039626	FJ039626
*C. camphoratus*	TRTC175623	Canada	PP383785	-
*C. caninus*	CFP627	Sweden	KX302250	-
*C. caninus*	HMJAU44372	China	OP620657	OP620671
*C. cf. cotoneus*	HMAS254210	China	KX513580	-
*C. cf. cotoneus*	HMAS260331	China	KX513578	-
*C. cf. cotoneus*	QL060	China	HM105543	-
*C. cf. cotoneus*	ZWL560	China	KX444284	-
*C. cinnamomeus*	OS480	Norway	KC842413	KC842483
*C. cinnamomeus*	UBCF19609	Canada	HQ604650	HQ604650
*C. cotoneus*	19XML11153	China	OP620655	OP620666
*C. cotoneus*	CFP1032	Sweden	MW009216	-
*C. cotoneus*	OS579	Norway	KC842423	KC842493
*C. cotoneus*	PML5260	France	MW010117	-
*C. cotoneus*	PML5429	France	MW010116	-
*C. cruentoides*	JAC13529	New Zealand	MW263695	-
*C. cruentoides*	PDD101864	New Zealand	KJ635217	KJ635217
*C. delibutus*	F17048	Canada	FJ717515	FJ717515
*C. dysodes*	PDD70499	New Zealand	GU233340	-
*C. dysodes*	PDD72664	New Zealand	MH101614	MH108334
*C. epsomiensis*	HMJAU44505	China	ON254423	OR105108
*C. epsomiensis*	KM74963	United Kingdom	MK010952	-
*C. ferrugineifolius*	IBMMoser19910305	North America	NR171327	-
*C. ferrugineifolius*	SHLindstromCFP969	North America	MT935278	-
*C. ferrusinus*	JB810613	Spain	KY657254	-
*C. ferrusinus*	JB888116	Spain	KY657255	-
*C. fibrillososalor*	MHHNU32070	China	OR660685	-
*C. fibrillososalor*	MHHNU32494	China	OR647481	-
*C. flammeouraceus*	H6029919	North America	NR170035	-
*C. flammeouraceus*	HMJAU60648	China	OL891470	-
*C. fusisporus*	BILAS51540	Lithuania	ON261481	-
*C. fusisporus*	BILAS51600	Lithuania	ON406294	-
***C. griseoaurantinus***	**HMAS 353381**	**China**	**PQ796783**	**PV174519**
***C. griseoaurantinus***	**HMAS 353382**	**China**	**PQ796784**	**PV174520**
*C. hughesiae*	JFA13086	USA	MW009224	-
*C. hughesiae*	TENN068689	USA	MW009225	-
*C. illibatus*	HMJAU48760	China	MW911735	OP620668
*C. illibatus*	iNat13972929	USA	OK346478	-
*C. indotatus*	PDD88257	New Zealand	KJ421110	KJ421110
*C. indotatus*	PDD92040	New Zealand	GU222322	-
*C. liyui*	HMJAU58938	Jilin, China	OP620661	-
*C. liyui*	HMJAU58939	Jilin, China	OP620660	OP620672
*C. luhmannii*	TUB019808	Germany	KJ421111	KJ421111
*C. luhmannii*	TUB019811	Germany	KJ421114	KJ421114
*C. pseudocamphoratus*	HMJAU48698	China	OM001483	OM001524
*C. pseudocamphoratus*	HMJAU48798	China	OM001489	-
*C. pseudosalor*	MHHNU32082	China	OR660686	-
*C. pseudosalor*	MHHNU32148	China	OR660688	OR647505
*C. pseudosalor*	MHHNU8349	China	OR647352	-
*C. putorius*	TNO7411HT	USA	KR011124	-
*C. rotundisporus*	PDD72611	New Zealand	AY669612	AY669612
*C. rotundisporus*	PDD96298	New Zealand	MH101550	-
*C. saginus*	IB19960705	USA	AF325608	-
*C. saginus*	T30	Norway	KC842448	KC842518
*C. salor*	TUB011838	Germany	AY669592	AY669592
*C. selinolens*	FR2013185	Tunisia	MW010072	-
*C. selinolens*	MPU1116858	France	MW010172	-
*C. sommerfeltii*	HMJAU44457	China	OP620652	OP620663
*C. sommerfeltii*	SOMF30854	Spain	OQ398585	-
*Cortinarius* sp.	MEL2089705	Australia	GQ890326	JN940245
*Cortinarius* sp.	SWUBC741	Canada	DQ481671	-
*Cortinarius* sp.	T21468	China	OP620656	-
*C. spilomeus*	CFP1137	Sweden	KX302267	-
*C. spilomeus*	H6031514	Finland	KX302264	-
*C. spilomeus*	TUB011523	Europe	AY669654	AY669654
*C. subargyronotus*	C358	Hungary	OP099768	-
*C. subargyronotus*	H7018127	Finland	NR131871	-
*C. subcotoneus*	AB08-10-331	France	MW010167	-
*C. subcotoneus*	GS15	Germany	MW010106	-
*C. subcotoneus*	PML2143	France	MW010122	-
*C. subsalor*	HMJAU48758	China	MW911733	-
*C. subsalor*	HMJAU48759	China	MW911734	OP620670
*C. subsanguineus*	HMAS250503	China	MK411450	-
*C. subsanguineus*	HMJAU48961	China	OP620653	OP620664
*C. subtortus*	F16111	North America	FJ157044	FJ157044
*C. subtortus*	TUB011382	Europe	AY174857	AY174857
*C. subtropicus*	MHHNU31981	China	OR660687	OR647502
*C. subtropicus*	MHHNU33533	China	OR647488	-
*C. tabularis*	CFP949	Sweden	KX302275	-
*C. tabularis*	H7022440	Finland	KX302279	-
*C. tasmacamphoratus*	HOA20606A0	Tasmania	AY669633	AY669633
*C. tessiae*	PDD107517	New Zealand	MG019356	MG019365
*C. tetonensis*	JFA10350	North America	MZ580436	-
*C. tibeticisalor*	HMJAU48763	China	MW911730	-
*C. tibeticisalor*	HMJAU48764	China	MW911729	OP620669
*C. uliginosus*	KH7	Norway	KC842412	KC842482
*C. uliginosus*	TUB011823	Germany	AY669584	AY669584
*C. umbrinolens*	NFSG20231021	Britain	PP355760	-
*C. umbrinolens*	TUB011918	Germany	AY669658	AY669658
*C. veronicae*	JAC10781	New Zealand	MW263653	-
*C. veronicae*	PDD68468	New Zealand	KC017355	-
*C. viridipileatus*	OTA61977	New Zealand	MK546592	MK546595
*C. viridipileatus*	OTA64087	New Zealand	MK546593	NG228804
*C. xiaojinensis*	HMAS274355	China	MK411447	-
*C. xiaojinensis*	HMJAU58895	China	OP620654	OP620665
*C. yadingensis*	HMAS254811	China	OR538889	-
*C. yadingensis*	HMAS254819	China	OR538892	-
*C. yadingensis*	HMAS254820	China	OR538893	-
*C. yadingensis*	HMAS280697	China	OR538890	-
*C. yadingensis*	HMAS280698	China	OR538891	-
***C. yonganensis***	**HMAS 353379**	**China**	**PQ796785**	**PV174521**
***C. yonganensis***	**HMAS 353380**	**China**	**PQ796786**	**PV174522**
*Hebeloma eburneum*	ue2089	England	JN943880	JN939950
*H. alpinum*	ue3984	Greenland	JN943859	JN939969

New species described herein are highlighted in bold (*C.*, *Cortinarius*).

## 3 Results

### 3.1 Phylogenetic analyses

**Figure 1:**
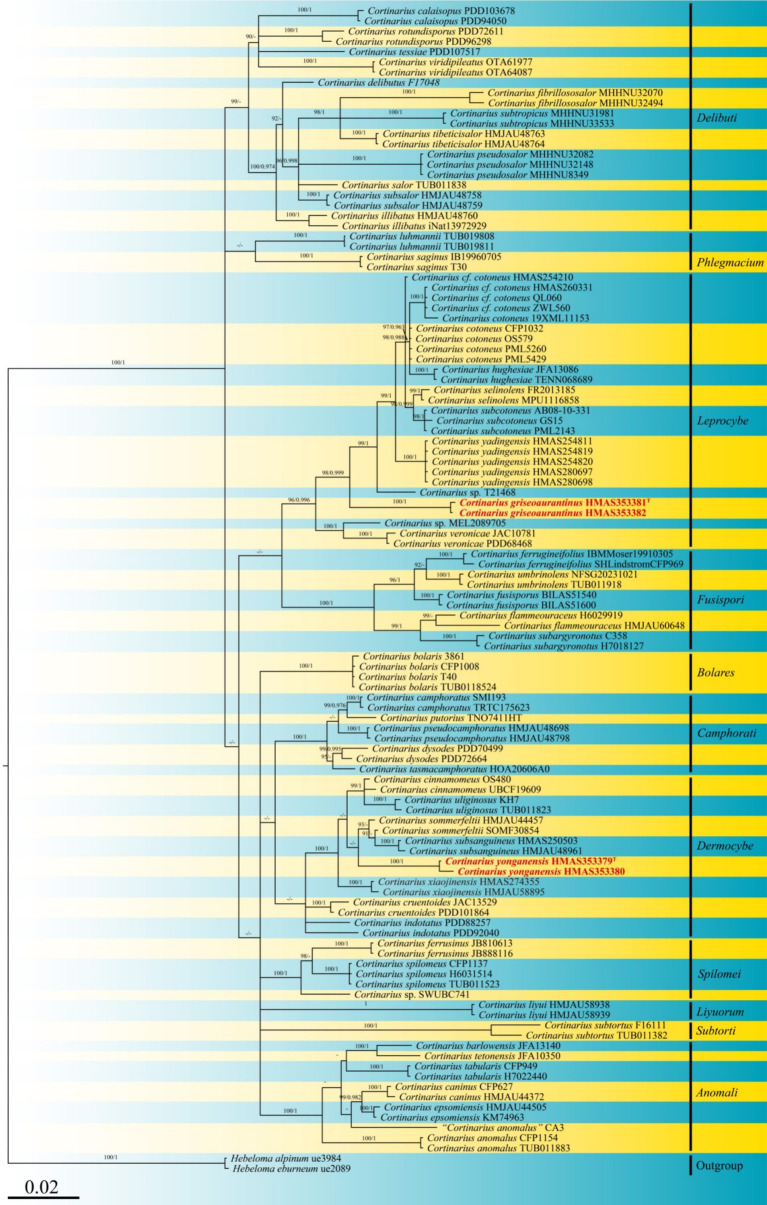
Phylogeny inferred from ITS and nrLSU sequences using Bayesian analysis. Bootstrap values (ML/BI) above 90/0.95 are indicated above the individual branches. The scale bar represents the number of nucleotide substitutions per site. New species are highlighted in red and bold. Arrows indicate the support values at the branching points. The superscript “T” denotes the type strain of the new species.

Four sequences were generated from the specimens, with two corresponding to the ITS locus and two to the nrLSU locus, and these were deposited in GenBank (see accession numbers in Table 1). To construct a robust phylogenetic tree, homologs were identified through initial BLAST searches, leading to a final DNA sequence alignment comprising a dataset of 114 specimens. After removing ambiguously aligned regions and gaps, the final combined sequence data matrix comprised 3,967 characters. Sequences from two specimens of *Hebeloma cylindrosporum* (IK-H0467 and HKAS134461) served as the outgroup. The tree topology derived from Maximum Likelihood (ML) analysis was similar to that obtained from Bayesian Inference (BI) analysis (refer to Figure 1). The best scoring maximum-likelihood (ML; ILn = −16999.896), based on RAxML analysis of the combined ITS-nrLSU dataset, is presented.

Phylogenetically, *Cortinarius griseoaurantinus* and *Cortinarius yonganensis* were separated into individual lineages with strong statistical support and were distinct from their closest taxa. *C. griseoaurantinus* nested within a clade of the subgenus *Leprocybe*, clustering alongside *C. veronicae* and *C. yadingensis*. *C. yonganensis* nested within a clade of the subgenus *Dermocybe*, clustering alongside *C. subsanguineus* and *C. sommerfeltii*.

### 3.2 Taxonomy

**Figure 2:**
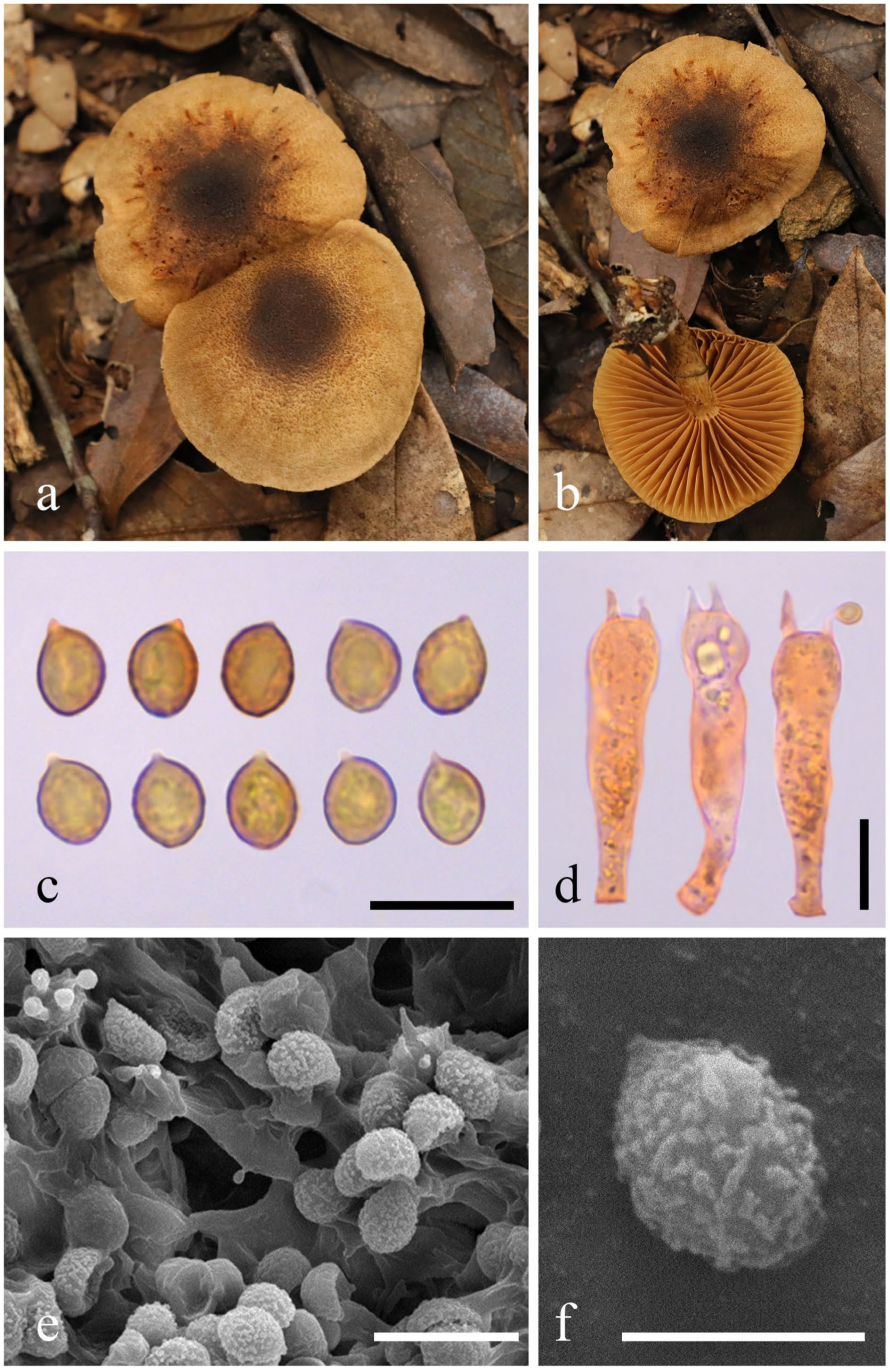
Morphological characteristics of *Cortinarius griseoaurantinus* (HMAS 353381). **(a,b)** Basidiomata; **(c)** Basidiospores in Congo Red reagent; **(d)** Basidium in Congo Red reagent; **(e,f)** SEM images of basidiospores. Bars: **(c,d,e)** = 10 μm; **(f)** = 5 μm.

**Figure 3:**
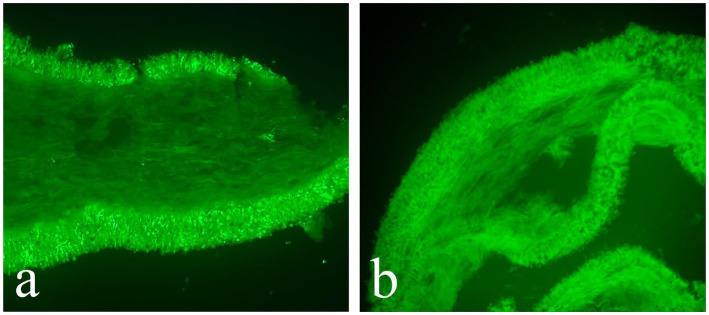
Fluorescence reactions when exposed to ultraviolet light. **(a)** *Cortinarius griseoaurantinus* (HMAS 353381); **(b)** *Cortinarius yonganensis* (HMAS 353379).

***Cortinarius griseoaurantinus*** M. J. Zhu & Jun Z. Qiu, sp. nov. (Figures 1, 2 and 3)

Mycobank No.: 857170

Etymology: “griseo-” comes from “griseus,” meaning gray; “aurantinus” means orange, which is the color of the fungal pileus.

Diagnosis: The diagnosis indicates that this species, related to *C. yadingensis,* mainly differs in having a bright orange to brownish-orange pileus.

Holotype: CHINA, Fujian Province, Mingxi, Xiafang Township, Gaoyang Village, in a mixed forest at 26°33′43″N, 116°53′38″E, at an elevation of 499 m, collected on 3 September 2023 (holotype HMAS353381).

Description: Basidiomata are medium-sized. The pileus measures 33–55 mm in diameter, initially convex, expanding to become applanate at maturity, dry, and bright orange (5A5) to brownish orange (5C5), with a lighter color appearing as pale yellowish white (4A2) toward the margin; the disc ranges from slightly to more strongly brownish, featuring light brown (5D6) to brown (5F6); the surface is light brown (5D6) and ciliated; the margin is slightly striate with a light brown (5D6) hue; the context is 1–3 mm thick above the stipe. Lamellae are adnate to slightly adnexed, sub-sparse, and forking near the margin, presenting a light pastel yellow (3A4) to rotgold brownish orange (6C6) coloration. The stipe measures 3.5–5 × 0.6–0.8 cm, central, equal to slightly tapering upwards, with a base measuring 1–1.2 cm in diameter, dry, and displaying the same light pastel yellow (3A4) to rotgold brownish orange (6C6) background color as the lamellae. It is covered with light brown (5D6) to brown (5F6) fibrillose squamules, featuring an annular zone from the partial veil, remaining unchanging when bruised, hollow in the center, and soft. The spore print is brown. The odor is indistinct. The fluorescence reaction exhibits a bright green light under ultraviolet light.

Basidiospores (5.9) 6.1–6.6–7.5 (8.2) × (4.6) 4.9–5.3–5.9 (6.1) μm, Q = 1.23 ± 0.07 from a sample size of 40; they are subglobose, yellow-brown, and distinctly verrucose, often appearing punctate or aligned in lines. Basidia (30.7) 31.4-34.1-37.9 (42.0) × (6.9) 7.2-8.1-9.3(10.1) μm, Q = 4.26 ± 0.39 based on 20 samples, clavate, thin-walled, mostly subhyaline, with four sterigmata measuring (1.8) 2.2–3.1–4.4 (4.4) μm in length. The edges of the lamella display heterogeneity, comprising sterile hyphae that measure 10–20 × 6–8 μm, which are clavate, subhyaline, and thin-walled. Pleurocystidia are not present. The pileipellis features a well-developed epicutis, hyphae 6–10 μm wide, subcylindrical, colorless to brownish, thin-walled, and smooth; the hypodermium is also present, consisting of hyphae 13–20 μm in width, presenting irregularities in 5% KOH. Clamp connections are also present.

Additional specimens examined: China, Fujian Province, Huangxizhou, Wuyishan National Park, in a mixed forest located at 27°42′40″N, 117°45′36″E, at an elevation of 689 m, on 7 September 2023 (paratype HMAS 353382).

Habitat: This species is solitary in coniferous and broadleaf mixed forests. It was collected in September 2023 and occurs at an altitude range of 450–700 m, growing on the ground near Fagaceae trees.

Note: *Cortinarius griseoaurantinus* can be distinguished from other species of subgenus *Leprocybe* by its bright orange to brownish-orange pileus, with the disc varying from slightly to deeply brownish, and by its distinctly verrucose basidiospores (6.1–7.5 × 4.9–5.9 μm).

**Figure 4:**
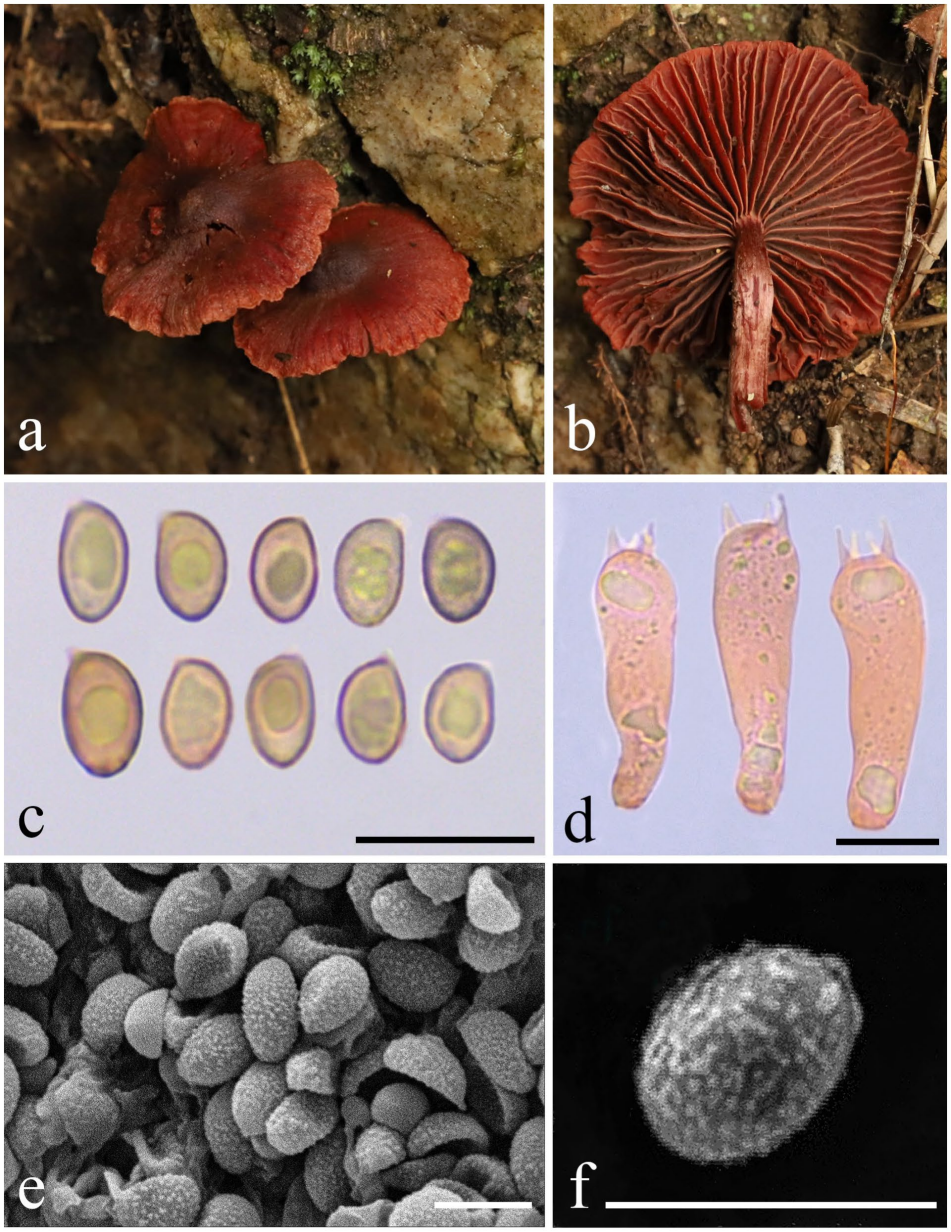
Morphological characteristics of *Cortinarius yonganensis* (HMAS 353379). **(a,b)** Basidiomata; **(c)** Basidiospores in Congo Red reagent; **(d)** Basidium in Congo Red reagent; **(e,f)** SEM images of basidiospores. Scale bars: **(c,d)** = 10 μm; **(e,f)** = 5 μm.

***Cortinarius yonganensis*** M. J. Zhu & Jun Z. Qiu, sp. nov. (Figures 1, 3 and 4)

Mycobank No.: 857171

Etymology: The term *“yonganensis*” is derived from Chinese and refers to Yong’an City in Fujian Province, China, which is the locality of the type collection.

Diagnosis: Its diagnosis differs from that of the related species *C. subsanguineus* primarily due to the presence of punctate basidiospores and a pileus featuring a prominent violet-brown umbo at the center, along with striations along the margin.

Holotype: CHINA, Fujian Province, Sanming City, Yong’an City, Nanxi, discovered in a mixed forest at coordinates 25°31′48″N, 117°13′48″E, at an elevation of 1,031 m, on 24 June 2023 (holotype HMAS 353379).

Description: Basidiomata are small to medium-sized. The pileus measures 20–40 mm in diameter, initially hemispherical, then convex, and finally applanate with a conspicuous umbo at the center; the margin is decurrent to straight, and the surface ranges from reddish-pink (9B5) to deep jasper red (9B7), featuring persistent violet-brown (10F6) minute floccose squamules from the universal veil; the violet-brown (10F6) umbo at the center is notable, and the margin is pale pink with orange to graurot (7B4) and antiviolent brown (10E7) striations; the context is 0.5–2 mm thick above the stipe. Lamellae are adnate to emarginate-adnate, sub-sparse, and forking with small branches near the margin, with color ranging from reddish pink (9B5) to deep jasper red (9B7). The stipe measures 2.0–5.0 × 0.3–0.5 cm, central, cylindrical, dry, and rust-red, covered with rust-red floccules from the universal veil; it is rough, solid to soft, and hollow in the center. The spore print is not recorded. The odor is indistinct. The fluorescence reaction under ultraviolet light is pale green.

Basidiospores (5.0) 5.3–6.0–6.6 (7.4) × (3.9) 4.1–4.3–4.6 (4.7) μm, Q=1.41 ± 0.11 from a sample size of 40, subglobose, yellow, and punctate. Basidia (22.1) 24.3–26.5–29.2 (30.0) × (7.0) 7.0–8.2–9.3 (9.9) μm, Q=3.27 ± 0.27 from a sample size of 20, clavate, thin-walled, mostly subhyaline, with four sterigmata, measuring (2.2) 2.4–3.1–3.6 (3.7) μm in length. The lamella edges exhibit heterogeneity, characterized by sterile hyphae measuring between 14–21×5.5–7 μm, which are clavate, subhyaline, and thin-walled. Pleurocystidia are absent. The pileipellis features a well-developed epicutis with hyphae measuring 5.5–7.5 μm in width, subcylindrical, colorless to brownish, thin-walled, and smooth. The hypodermium is present, with irregular hyphae that are 18.5–40 μm wide in 5% KOH. Clamp connections are also present.

Additional specimens examined: China, Fujian Province, Sanming City, Yong’an City, Nanxi, located in a mixed forest at 25°31′43″N, 117°13′46″E, at an elevation of 1,020 m, on 24th June 2023 (paratype HMAS 353380).

Habitat: This species is solitary in mixed forests. It was collected during the summer months (June to August) and is found at altitudes above 1,000 m, growing on rocky cliffs in mixed forests.

Note: *Cortinarius yonganensis* can be distinguished from other species of subgenus *Dermocybe* by its reddish-pink to deep jasper red pileus, which features a prominent violet-brown umbo at the center and a margin with violet-brown striations. Additionally, the basidiospores (measuring 5.3–6.6 × 4.1–4.6 μm) appear punctate.

## 4 Discussion

In this study, we identified two new taxa of *Cortinarius*: *C. griseoaurantinus* and *C. yonganensis*. The identification of these species was based on molecular phylogenetic analyses of the ITS and nrLSU loci, which demonstrated the separation of *C. griseoaurantinus* and *C. yonganensis* from their closest relatives, along with their distinct morphological characteristics. Additionally, we utilized electron microscopy to capture photographs of the surface ornamentation of spores, providing further features for differentiating between the species. The morphology of the two new species aligns well with the subgenus concept. For example, the basidiomata of *C. griseoaurantinus* are medium-sized, with a distinctly orange pileus. In contrast, *C. yonganensis* features a brightly colored pileus ranging from reddish-pink to deep jasper red, accompanied by a relatively slender stipe measuring 2.0–5.0 × 0.3–0.5 cm.

According to the phylogram, *C. griseoaurantinus* lies within the subgenus *Leprocybe* and clusters with *C. veronicae* and *C. yadingensis*. All three species produce medium-sized basidiomata, with pileus diameters ranging from 5 to 6 cm (*C. veronicae*), 2 to 6 cm (*C. yadingensis*), and 3.3 to 5.5 cm (*C. griseoaurantinus*), and stipes of comparable lengths (3.5 to 8 cm for *C. yadingensis* and 3.5 to 5 cm for *C. griseoaurantinus*). However, *C. yadingensis* has a pale brown to rust brown pileus with fibrillose squamules, while *C. veronicae* features a red-orange to brick-red pileus and lamellae (Soop, 1998). In contrast, as mentioned, *C. griseoaurantinus* has a bright orange to brownish-orange pileus with the disc slightly to more strongly brownish. Furthermore, *C. yadingensis* has a brownish stipe with fibrillose squamules, whereas *C. veronicae* has a pink to yellowish-pink stipe with cinnabar-orange girdles, and *C. griseoaurantinus* has a pastel yellow to rot-gold brownish-orange stipe (Hong et al., 2024).

*Cortinarius yonganensis* was found to cluster within the subgenus *Dermocybe,* being related to *C. subsanguineus* and *C. sommerfeltii*. In particular, *C. subsanguineus* is both morphologically and phylogenetically closely related to *C. yonganensis*. However, *C. subsanguineus* generally exhibits larger, ellipsoid, verrucose basidiospores (6.5–8 × 4.5–5.5 μm), whereas *C. yonganensis* has smaller, subglobose, punctate basidiospores (5.3–6.6 × 4.1–4.6 μm). Both species have similar pileus colors, but *C. yonganensis* differs from *C. subsanguineus* by having a conspicuous violet-brown umbonate at the center and a margin with violet-brown striations (Yuan et al., 2020). Morphologically, *C. sommerfeltii* also differs from *C. yonganensis* with its deep reddish-brown to ochraceous-brown pileus, while *C. yonganensis* has a reddish-pink to deep jasper red pileus with a conspicuous violet-brown umbo at the center and a margin marked by violet-brown striations and punctate details. Additionally, *C. sommerfeltii* is commonly found in coniferous forests (Høiland, 1983), whereas *C. yonganensis* is found on rocky cliffs in mixed forests.

In summary, we describe two new species of *Cortinarius* through the integration of morphological traits, molecular phylogenetic analysis, and ecological information. The identification of *Cortinarius griseoaurantinus* (subgenus *Leprocybe*) and *Cortinarius yonganensis* (subgenus *Dermocybe*) enriches our understanding of the genus *Cortinarius* and its ecological relationships. As species of *Cortinarius* have been rarely discovered or characterized in China, our data suggest the potential for additional discoveries and contribute to a better understanding of the evolution, origin, and geographical distribution of this important group of mushrooms.

## Data Availability

The datasets presented in this study can be found in online repositories. The names of the repository/repositories and accession number(s) can be found in the article/supplementary material.

## Footnotes

^1^ http://blast.ncbi.nlm.nih.gov
